# Normal delivery following use of galcanezumab until the first and second trimesters of pregnancy: A report of 2 cases

**DOI:** 10.1097/MD.0000000000047895

**Published:** 2026-02-28

**Authors:** Hisanao Akiyama, Yoshihisa Yamano

**Affiliations:** a Department of Neurology, St. Marianna University School of Medicine, Kawasaki, Kanagawa, Japan.

**Keywords:** CGRP-related antibody drug, first trimester, galcanezumab, migraine without aura, pregnancy, second trimester

## Abstract

**Rationale:**

Clinical trials have demonstrated the efficacy, rapid action, and safety of calcitonin gene related peptide-related antibody drugs for migraine prevention, and subcutaneous galcanezumab was launched in Japan in April 2021. However, the safety of this drug in pregnant migraine patients has not been demonstrated in humans, with evidence of safety available only from animal studies. We report 2 cases in which subcutaneous galcanezumab was administered continuously until the first and second trimesters of pregnancy, resulting in normal births without any complications for either the mother or child.

**Patient concerns:**

The cases involved pregnant patients, aged 34 and 43, with long-standing migraines without aura.

**Diagnoses:**

Migraines without aura.

**Interventions:**

They were administered galcanezumab until the fifth week and fifth month of pregnancy, respectively. After discontinuation of treatment, the increase in the frequency of the mothers’ migraines remained mild.

**Outcomes:**

Each mother safely gave birth to a baby boy, with acetaminophen administered as needed for the management of headaches. Subsequent checkups showed that both mothers and their children were healthy.

**Lessons:**

The safety of galcanezumab administration in pregnant women has only been reported in scattered case reports, and further case studies are needed. It is considered necessary to continue to monitor newborns for the development of neurological abnormalities, including higher brain functions.

## 1. Introduction

Calcitonin gene related peptide (CGRP)-related antibody drugs, 3 of which have launched in Japan since April 2021, are highly effective, fast-acting, and safe for migraine prevention,^[1-13]^ and these medications are gradually replacing traditional migraine preventive medications such as antihypertensives, antiepileptics, and antidepressants.^[14]^ While the majority of these conventional preventive medications are contraindicated for use in pregnant women, the safety of CGRP-related antibody drugs in pregnant or nursing women remains unclear. Animal studies have shown fetal transfer but no adverse effects on the fetus.^[15]^ Therefore, the package inserts clearly state that they can be administered to pregnant women or women who may be pregnant if the therapeutic benefits are deemed to outweigh the risks. However, to date, there have been few reports on the safety of administering CGRP-related antibody drugs such as galcanezumab, fremanezumab, and erenumab, which are available in Japan, to pregnant and nursing women, who often overlap with the age group most susceptible to migraine.^[16-27]^

Here, we report 2 cases in which galcanezumab was administered continuously until the first and second trimesters of pregnancy, resulting in normal births. We believe these cases are valuable for demonstrating that galcanezumab can be used safely and effectively during pregnancy.

## 2. Case presentations

### 2.1. Case 1

The patient was a 34-year-old female obstetrician. Since high school, at age of 16 years, she had experienced headaches approximately once a month, but managed them by staying in bed without taking any medication. In 2010, when she was 22 years old and attending university, she developed headaches that lasted for 2 days every weekend. She visited our hospital and was diagnosed with migraine without aura according to the International Classification of Headache Disorders. She had previously used lomerizine hydrochloride, valproic acid, amitriptyline, and Chinese herbal medicines as preventive medications. Along with these preventive drugs, acute medications such as sumatriptan nasal spray or self-subcutaneous injection, zolmitriptan, rizatriptan, acetaminophen, and nonsteroidal anti-inflammatory drugs were also used but were ineffective. She ultimately relied solely on the acute medication naratriptan. At age 24 years, she became a medical resident and her migraine frequency increased to approximately 15 days per month, but she continued to take naratriptan alone as needed. Then, in 2018, she became pregnant at the age of 30 years with her first child. Her headaches increased from around the 10th week of pregnancy, and she successfully delivered the baby while managing headaches with acetaminophen as needed. After giving birth, migraines began to recur around the time her period resumed, and she was treated with naratriptan as needed.

**Figure 1.**
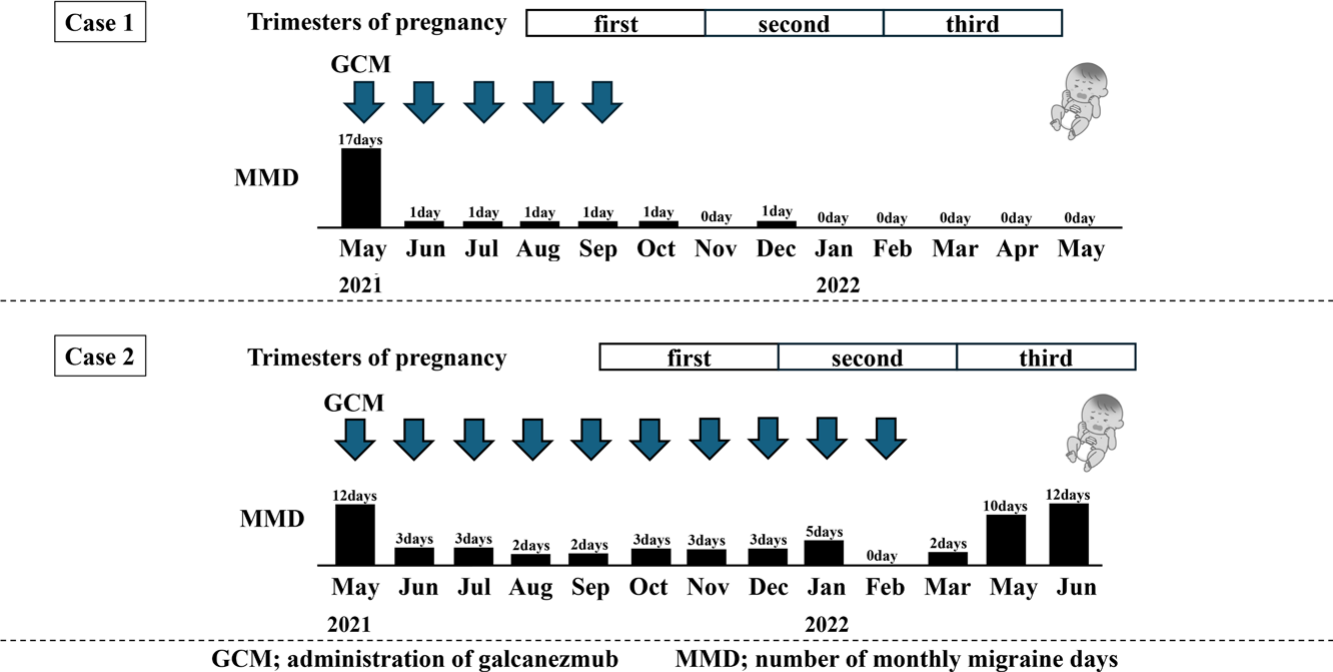
In cases 1 and 2, galcanezumab was administered until the fifth week and fifth month of pregnancy, respectively, and both mothers gave birth to healthy boys. Physical examination at birth revealed no notable abnormalities or obvious external malformations. GCM = administration of galcanezmub, MMD = number of monthly migraine days.

Galcanezumab was launched in Japan in April 2021 (when the patient was 34 years old). She was taking 20 naratriptan tablets for 17 migraine days per month at the time of her regular checkup on May 7, and was immediately switched to subcutaneous galcanezumab on the same day. Because the frequency of migraines subsequently decreased to only 1 day per month and the treatment showed remarkable efficacy, she continued galcanezumab. However, on September 24, 4 months after starting galcanezumab, she reported that she was pregnant. She informed the doctor that although she would prefer to discontinue the medication, she had been traumatized by the severity of the migraines she experienced during her previous pregnancy and she feared the headaches would make it impossible for her to continue practicing medicine. She therefore declined to discontinue galcanezumab, and her spouse, a neurologist, also agreed with continuing the medication. A dose of galcanezumab was administered that same day (at 5 weeks and 6 days of pregnancy). However, 1 month later at her regular consultation on October 22 (5 months after the start of galcanezumab), the patient again expressed concern about the effects of galcanezumab on the fetus and requested discontinuation of the drug, which was discontinued that day. Fortunately, after discontinuation of the drug, the frequency of migraines did not increase (number of monthly migraine days (MMDs) was 0–1), and treatment was provided with acetaminophen 1000 mg per administration. Both the mother and fetus remained in good health, and on May 24, 2022 (40 weeks and 3 days of gestation), a baby boy was delivered via a painless vaginal delivery. At birth, a physical examination of the baby revealed the following: height, 51.0 cm; weight, 3425 g; chest circumference, 32.0 cm; and head circumference, 35.5 cm, with no notable abnormalities or obvious external malformations (Fig. 1). The baby was subsequently breastfed for 1 year and 5 months, and there have been no notable abnormalities (at 1.5 years old, his height was 76.8 cm and weight was 9.4 kg; at 3 years old, his height was 88.0 cm and weight was 12.2 kg) in his growth or intellectual development. After weaning, migraines recurred about 8 days a month, and galcanezumab was readministered 2 years after childbirth. After readministration, migraines decreased to about 0 to 2 days per month, and galcanezumab was effective.

### 2.2. Case 2

A 43-year-old female office worker began having headaches at age 17 years and was diagnosed with migraines without aura. Prior to the birth of her first child, her headaches were well controlled with the administration of triptans. However, 4 months after giving birth, at age 37 years, she began to reduce the frequency of breastfeeding, and her menstruation resumed. Due to the combined effects of a busy schedule, stress, and fatigue from work and childcare, the severity and frequency of her migraines worsened, leading to chronic migraines that required her to be transported to an emergency room by ambulance. She began taking various migraine preventive medications, including lomerizine hydrochloride, valproic acid, amitriptyline, propranolol, and traditional Chinese herbal medicine. However, the migraines proved resistant to treatment with these medications, forcing her to temporarily take a leave of absence from work. Despite the use of various migraine preventive medications, her headache frequency remained at 2 to 3 d/wk (MMDs was 12). She discontinued all previous preventive medications on May 14, 2021, at age 42 years. She also began subcutaneous galcanezumab on the same day. One month after the initiation of these medications, her migraine frequency significantly decreased to 0 to 3 d/mo.

At a routine visit 7 months after starting galcanezumab (at age 43 years), on December 3, 2021 (3 months of gestation), the woman reported being pregnant. Although discontinuing galcanezumab was suggested, the severity and pain of her migraines had traumatized her and she did not wish to discontinue the medication. Her spouse also wanted to respect her opinion, so galcanezumab was continued until February 4, 2022 (9 months after starting the medication and at 5 months of gestation), when the obstetrician persuaded her to discontinue the medication. After discontinuing the medication, the frequency of her migraines significantly increased to 3 d/wk (MMDs was 10 to 12), but this was managed with acetaminophen 1000 mg per administration. Both the mother and fetus remained in good health, and a baby boy was delivered via a painless vaginal delivery on June 24, 2022 (38 weeks and 4 days of gestation). At birth, a physical examination of the newborn revealed the following: weight, 2909 g; height, 50.5 cm; chest circumference, 32.0 cm; and head circumference, 33.0 cm, with no notable abnormalities or obvious external deformities (Fig. 1). The baby was subsequently breastfed for 2 months, and physical examination at the 3-month checkup (October 17) revealed the following: weight, 6465 g; height, 63.4 cm; chest circumference, 41.6 cm; and head circumference, 41.0 cm. At the 4-month checkup (November 21) the baby weighed 7165 g, was 66.8 cm tall, had a chest circumference of 43.5 cm, and had a head circumference of 42.2 cm, at the 1-year checkup, weight, 8.7 kg; height, 76.5 cm, at the 2-years checkup, weight, 11.2 kg; height, 83.2 cm, at the 3-years checkup, weight, 13.6 kg; height 94.2 cm with no his growth or intellectual developmental abnormalities noted. After weaning, migraines recurred about 5 days a month, and galcanezumab was readministered 5 months after childbirth. After readministration, migraines decreased to about 1 to 2 d/mo, and galcanezumab was effective.

## 3. Discussion

Although the majority of migraine patients experience a decrease in migraine attack frequency during pregnancy and rarely require preventive medication,^[28-31]^ clinical practice occasionally encounters patients whose migraine frequency does not decrease during pregnancy. In such cases, the fetal organogenesis period (weeks 4–12 of pregnancy) is particularly problematic, and the administration of CGRP-related antibody drugs, which have a long half-life, is considered to be an issue. Meanwhile, it has been reported that maternal IgG increases in fetal blood from the early second trimester, with most of this IgG transferred to the third trimester.^[32]^ However, there are currently no reports on whether CGRP-related antibody drugs, which are IgG4 agent with high placental transfer, exhibit similar transfer kinetics. Therefore, this case of galcanezumab administration during the first or second trimester of pregnancy is considered to be clinically valuable.

Currently, 3 subcutaneous formulations of CGRP-related antibody drugs are available in Japan: galcanezumab, fremanezumab, and erenumab. Randomized controlled trials have demonstrated the efficacy, rapid action, and safety of all drugs for migraine prevention.^[1-13]^ However, these trials did not include pregnant or nursing women, and safety data in humans are limited.^[1-13]^ Although no clear harm to the fetus has been confirmed in animal studies, the safety of CGRP-related antibody drugs in pregnant or nursing women is unknown owing to the possibility that human IgG may be transferred to the fetus via the placenta or to the newborn via breastfeeding.^[15]^ The accompanying instructions also state that the drug should only be administered to pregnant or potentially pregnant women if the therapeutic benefits are deemed to outweigh the risks, and that in nursing women, the continuation or discontinuation of breastfeeding should be considered, taking into account the therapeutic benefits and the benefits of breastfeeding. As a general rule, the use of these drugs by pregnant or nursing women is not recommended. Expert opinions of the Japanese Headache Society and European Headache Federation^[33]^ also recommend that drug suspension should be considered for patients who are or may become pregnant or who are planning to become pregnant.

Although previous database analyses and case reports from the World Health Organization (WHO) pharmacovigilance database and WHO VigiBase® have not reported an increased risk of congenital abnormalities or miscarriage due to CGRP-related antibody drugs, the number of cases was limited, and the long-term effects are unknown.^[16,17]^ In a report by Elosua-Bayes et al, no clear complications or serious effects in newborns were observed following the use of CGRP-related antibody drugs during pregnancy.^[18]^ In an analysis of a large US insurance database, Hoffman et al also reported that there are currently not enough data on the use of galcanezumab in pregnant women to compare it with that of other drugs.^[19]^ Furthermore, the use of fremanezumab and erenumab during pregnancy should generally be avoided, and if their use is unavoidable, careful expert judgment is required, taking into account the benefit-risk balance.^[18-27]^ Bonifácio et al also reported 3 cases of pregnancy after the discontinuation of erenumab. One patient had a spontaneous abortion due to gestational trophoblastic neoplasia, but a direct relationship to erenumab was unlikely. The other 2 patients both gave birth to full-term infants without complications, and the newborns were healthy. They concluded that further follow-up of pregnant women and newborns and the reporting of additional cases are important for safety evaluation.^[22]^ In an updated review published in 2025, Ghadiri-Sani et al also reported that, as a general rule, the use of CGRP-related antibody drugs should be avoided in migraine patients of childbearing age or during pregnancy, and that the risks and benefits should be considered on an individual basis.^[27]^

As such, there are many reports stating that the administration of CGRP-related antibody drugs, such as galcanezumab, fremanezumab, and erenumab, to pregnant or nursing women has not been shown to result in any major adverse events or significant increased risks to the fetus or newborn.^[21-27^ However, the data are limited, and long-term safety and rare adverse events remain unknown. Therefore, the prevailing opinion is that administration to pregnant or nursing women should be carefully considered.^[16-27]^

In the 2 cases reported here, galcanezumab was administered continuously until the first and second trimesters of pregnancy, but no abnormalities were observed in either the mothers or the newborns. Although some time had passed from the discontinuation of galcanezumab, the growth of the newborns during breastfeeding was good. In both cases, the continuation of galcanezumab administration was based on the consent of the pregnant woman and her spouse, and these cases are considered valuable for demonstrating that galcanezumab can be continued until early or mid-pregnancy. In addition to continuing to monitor the development of newborns, it is considered urgent to collect data globally and conduct clinical trials on the safety of CGRP-related antibody drugs in pregnant and nursing women, who are prone to migraines and require early intervention.

## Author contributions

**Data curation:** Hisanao Akiyama.

**Investigation:** Hisanao Akiyama.

**Project administration:** Hisanao Akiyama.

**Validation:** Hisanao Akiyama.

**Writing – original draft:** Hisanao Akiyama.

**Writing – review & editing:** Hisanao Akiyama, Yoshihisa Yamano.
